# The complete mitochondrial genome of the spiny red gurnard *Chelidonichthys spinosus* McClelland, 1844 (Scorpaeniformes: Triglidae)

**DOI:** 10.1080/23802359.2021.1889412

**Published:** 2021-03-18

**Authors:** Hui Jia, Hao Xu, Weiwei Xian, Yuquan Li, Hui Zhang

**Affiliations:** a School of Marine Science and Engineering, Qingdao Agricultural University, Qingdao, China; b CAS Key Laboratory of Marine Ecology and Environmental Sciences, Institute of Oceanology, Chinese Academy of Sciences, Qingdao, China; c Laboratory for Marine Ecology and Environmental Science, Qingdao National Laboratory for Marine Science and Technology, Qingdao, China; d Center for Ocean Mega-Science, Chinese Academy of Sciences, Qingdao, China; e Qingdao Marine Hazard Mitigation Service, Qingdao, China

**Keywords:** *Chelidonichthys spinosus*, marine fish, mitochondrial genome, NGS, spiny red gurnard

## Abstract

The spiny red gurnard, *Chelidonichthys spinosus* is a common marine economic fish species along the coast of China. In the present study, the complete mitochondrial genome of spiny red gurnard collected from the Yangtze Estuary was determined by next-generation sequencing (NGS). The mitogenome is a circular nucleotide 16,466 bp in length and has the typical vertebrate genome structure of 13 protein-coding genes, two ribosomal RNA genes, 22 transfer RNA genes, and two control regions (Origin of Heavy chain and Origin of Light chain). The termination associated sequence (TAS), the central conserved sequence block (central CSB), and the conserved sequence block (CSB) were detected in the control region. Phylogenetic analysis of *C. spinosus* places in a fully supported clade with *C. kumu* in a sister position to *Eutrigla gurnardus*. The complete mitochondrial genome sequence of the spiny red gurnard provides baseline genetic information for future studies on the molecular systematics and phylogeny of bony fishes in the *Triglidae*.

The spiny red gurnard *Chelidonichthys spinosus* is a tropical marine fish, mainly distributed in the Northwest Pacific from southern Hokkaido, Japan to the South China Sea (Zhang et al. 2009). It is a common commercial fish along the coast of China and the target of bottom trawl fishing (Wang et al. 2018). Recently, with the increase in fishing intensity, the population sizes of many economic fishes along the coast of China have declined (Li et al. 2017). Next-generation sequencing (NGS) has revolutionized the field of molecular biology through its high speed and ability to generate large amounts of genomic data (Schuster 2008; Koboldt et al. 2013). We used NGS technology to determine the complete mitogenome of *C. spinosus*, which could be used to provide insight into population processes and the evolutionary history of the spiny red gurnard (Zhang and Xian 2015).

The DNA was extracted from muscle tissue of a spiny red gurnard collected from station 33 (123E, 30.75N) of the Yangtze estuary in the August 2020 and deposited in the CAS Key Laboratory of Marine Ecology and Environmental Sciences, Institute of Oceanology, Chinese Academy of Sciences, and its catalog number is 4-M-M-5 (Yibang Wang, wangyibang@qdio.ac.cn). The sequencing was completed using the Illumina NovaSeq sequencing platform (Illumina, San Diego, CA). The genome sequence was assembled and analyzed using A5-miseq v20150522 (Coil et al. 2015) and SPAdes v3.9.0 (Bankevich et al. 2012). The pilon v1.18 (Walker et al. 2014) software was used to correct the results to obtain the final mitochondrial sequence. Annotation of the complete mitochondrial genome sequence was performed with the MITOS web server (http://mitos2.bioinf.uni-leipzig.de/index.py) (Bernt et al. 2013). The maximum-likelihood (ML) tree was constructed using the ML method withK2P distances in MEGA10 software with 1000 bootstrap replicates (Kumar et al. 2018).

The complete mitogenome of the spiny red gurnard was 16,466 bp in length (GenBank accession MW300429), similar to the size of other teleost mitogenomes. As in other vertebrates (Miya et al. 2001), it contained 13 protein-coding genes, two rRNA (12SrRNA and 16S rRNA), 22 tRNA, and two control regions (OH and OL). Most of the genes of spiny red gurnard were encoded on the N-strand, with only NAD 6 and eight tRNA (tRNA^Gln^, tRNA^Ala^, tRNA^Asn^, tRNA^Cys^, tRNA^Tyr^, tRNA^Ser^, tRNA^Glu^, and tRNA^Pro^) genes encoded on the J-strand. Among the 13 protein coding genes, ATP 6 and ATP 8 overlap by 10 nucleotides, and NAD 4 and NAD 4L share seven nucleotides. The NAD 5 and NAD 6 genes overlap by four nucleotides on the opposite strand. The ATG codon initiates 12 of the 13 protein coding genes (NAD 1, NAD 2, CO II, ATP 8, ATP 6, CO III, NAD 3, NAD 4L, NAD 4, NAD 5, NAD 6, and COB), and COI starts GTG. The stop codon TAA terminates nine genes (NAD 1, NAD 2, CO I, ATP 8, AYP 6, CO III, NAD 4L, NAD 5, and NAD 6), TAG terminates NAD 3, and the other genes for COII, NAD 4, and COB, are stopped with the incomplete T– by post-transcriptional polyadenylation (Ojala et al. 1981). The 12S and 16S ribosomal RNA genes of the spiny red gurnard comprise 946 bp and 1670 bp, respectively. They are located between tRNA^Phe^ and tRNA^Leu^, and are separated by tRNA^Val^, as they are in other vertebrates (Zhang et al. 2016). The 22 tRNA genes were interspersed in the genome and range in size from 65 to 74 bp and fold into cloverleaf secondary structures with normal base paring. The major non-coding region in the spiny red gurnard is located between tRNA^Pro^ and tRNA^Phe^, and is 515 bp in length. The termination associated sequence (TAS), central conserved sequence block (central CSB), and conserved sequence block (CSB), are detected in the control region and are similar to most bony fishes (Zhang et al. 2013).

Phylogenetic analysis of the complete mitogenome of *C. spinosus* fully resolved it in a clade with *C. kumu* (Figure 1). *Eutrigla gurnardus* occupied a sister position in the same clade. Kim et al. (2012) reported that the interspecific distances between *Hexagrammos sp.* and *C. spinosus* below 0.1% (Kim et al. 2012), which were also confirmed in our results.

**Figure 1.**
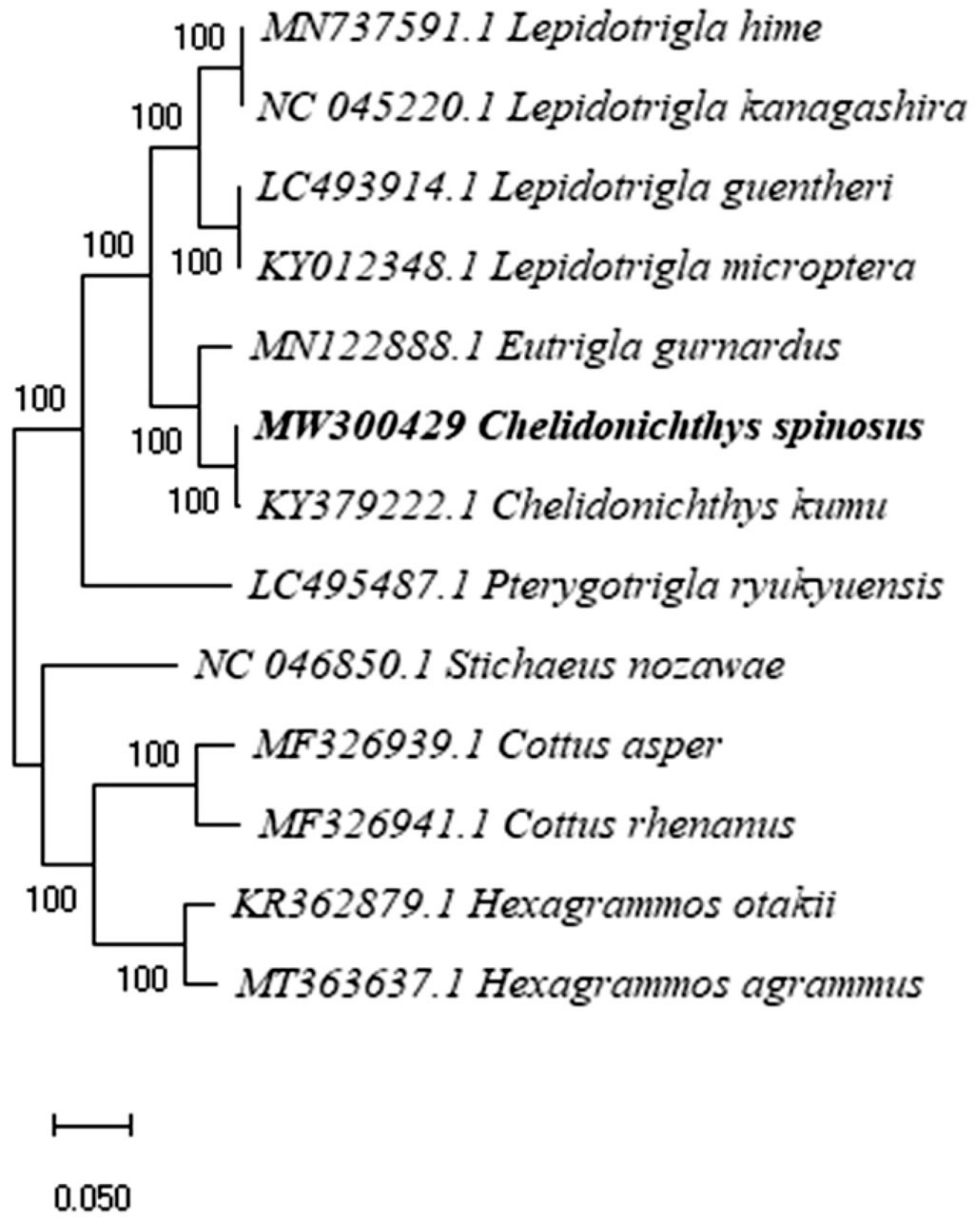
Maximum-likelihood tree of *Chelidonichthys spinosus* and 12 related Scorpaeniformes based on complete mitogenomes. Bootstrap values based on 1000 replicates and are represented at the nodes.

## Data Availability

Mitogenome data supporting this study are openly available in GenBank at nucleotide database, https://www.ncbi.nlm.nih.gov/nuccore/MW300429, Associated BioProject, https://www.ncbi.nlm.nih.gov/bioproject/PRJNA681041, BioSample accession number at https://www.ncbi.nlm.nih.gov/biosample/SAMN16931227, and Sequence Read Archive at https://www.ncbi.nlm.nih.gov/sra/SRR13153825.
